# T-Cell Responses to Immunodominant Listeria Epitopes Limit Vaccine-Directed Responses to the Colorectal Cancer Antigen, Guanylyl Cyclase C

**DOI:** 10.3389/fimmu.2022.855759

**Published:** 2022-03-09

**Authors:** John C. Flickinger, Jagmohan Singh, Yanki Yarman, Robert D. Carlson, Joshua R. Barton, Scott A. Waldman, Adam E. Snook

**Affiliations:** ^1^Department of Pharmacology & Experimental Therapeutics, Thomas Jefferson University, Philadelphia, PA, United States; ^2^Sidney Kimmel Cancer Center, Philadelphia, PA, United States; ^3^Department of Microbiology & Immunology, Thomas Jefferson University, Philadelphia, PA, United States

**Keywords:** listeria, vaccine, immunodominance, colorectal cancer, GUCY2C

## Abstract

The Gram-positive bacterium *Listeria monocytogenes* (Lm) is an emerging platform for cancer immunotherapy. To date, over 30 clinical trials have been initiated testing Lm cancer vaccines across a wide variety of cancers, including lung, cervical, colorectal, and pancreatic. Here, we assessed the immunogenicity of an Lm vaccine against the colorectal tumor antigen GUCY2C (Lm-GUCY2C). Surprisingly, Lm-GUCY2C vaccination did not prime naïve GUCY2C-specific CD8^+^ T-cell responses towards the dominant H-2K^d^-restricted epitope, GUCY2C_254-262_. However, Lm-GUCY2C produced robust CD8^+^ T-cell responses towards Lm-derived peptides suggesting that GUCY2C_254-262_ peptide may be subdominant to Lm-derived peptides. Indeed, incorporating immunogenic Lm peptides into an adenovirus-based GUCY2C vaccine previously shown to induce robust GUCY2C_254-262_ immunity completely suppressed GUCY2C_254-262_ responses. Comparison of immunogenic Lm-derived peptides to GUCY2C_254-262_ revealed that Lm-derived peptides form highly stable peptide-MHC complexes with H-2K^d^ compared to GUCY2C_254-262_ peptide. Moreover, amino acid substitution at a critical anchoring residue for H-2K^d^ binding, producing GUCY2C_F255Y_, significantly improved stability with H-2K^d^ and rescued GUCY2C_254-262_ immunogenicity in the context of Lm vaccination. Collectively, these studies suggest that Lm antigens may compete with and suppress the immunogenicity of target vaccine antigens and that use of altered peptide ligands with enhanced peptide-MHC stability may be necessary to elicit robust immune responses. These studies suggest that optimizing target antigen competitiveness with Lm antigens or alternative immunization regimen strategies, such as prime-boost, may be required to maximize the clinical utility of Lm-based vaccines.

## Introduction

Due to the unprecedented success of immune checkpoint inhibitors (ICIs) and adoptive cell therapy, immunotherapy has established itself as a pillar of cancer management alongside chemotherapy, surgery, targeted therapies, and radiotherapy (1, 2). While ICIs have been practice-changing, only 20% of patients respond to ICI therapy (3, 4). Notably, responsiveness to ICI therapy is associated with immunologically “hot” tumors characterized by significant immune cell infiltration (5, 6). In this context, there has been renewed interest in utilizing cancer vaccines as agents that expand tumor-specific T cells and promote T-cell infiltration into tumor microenvironments, working synergistically with ICI therapy (3, 4, 7). However, methods of cancer vaccination are highly variable, including peptide, nucleic acid, microbial-based, and others, with no consensus on optimal vaccine platforms (8). Therefore, a greater understanding of vaccine vector biology is urgently needed.

The Gram-positive bacterium *Listeria monocytogenes* (Lm) is an emerging platform for cancer immunotherapy. Lm is an attractive vector due to its tropism for antigen-presenting cells, leading to potent CD8^+^ T-cell immunity (9) and its ability to engage multiple aspects of the innate immune system and remodel immunosuppressive microenvironments (10, 11). Thus, multiple recombinant Lm vaccines secreting tumor antigens capable of inducing antitumor immunity have been explored (12). Moreover, attenuated Lm strains with favorable safety profiles have been developed for clinical testing. To date, over 30 clinical trials have been initiated testing attenuated Lm-based vaccines for cancers (13), originating from lung (14), cervix (15), colorectum (16), pancreas (17), and others.

Here, we examined the immunogenicity of an Lm-based vaccine expressing the colorectal cancer antigen guanylyl cyclase C (Lm-GUCY2C). Surprisingly, Lm-GUCY2C failed to prime GUCY2C-specific immune responses in mice, despite generating robust Lm-specific immunity. Studies revealed competition with immunodominant Lm-derived CD8^+^ T-cell epitopes as the underlying mechanism, which could be reversed by enhancing the MHC-binding affinity of GUCY2C-derived CD8^+^ T-cell epitopes. These studies reveal important mechanisms restricting the efficacy of Lm-based vaccines and novel approaches to Lm design and use to enhance that efficacy, particularly in the context of self/tumor-associated antigens.

## Materials and Methods

### Vaccines and Peptides

The live-attenuated double-deleted (LADD) strain of *Listeria monocytogenes* (Lm) containing deletions in virulence factors internalin B and actA (Δ*actA*Δ*inlB*) (18) was obtained from ATCC and served as the parental strain for all Lm vaccines in this study. Recombinant Lm-GUCY2C and Lm-LacZ were generated by gene synthesis of the codon-optimized mouse GUCY2C extracellular domain (GUCY2C_23-429_) or β-galactosidase_618-1024_, respectively, in-frame with a modified version of the first 100 amino acids of actA protein (called ActAN100* throughout) and the Syn18x5 enhancer sequence under control of the *actA* promoter (19). All other Lm constructs were similarly cloned. The genetic sequence was cloned into the pPL2 plasmid (kindly provided by Richard Calendar, UC Berkeley) and integrated into the Lm chromosome as previously described (20). Successful integration was confirmed by DNA sanger sequencing. Recombinant Lm was grown in brain-heart infusion (BHI) broth (Fisher Scientific) to OD_600_ of about 1.0 and stored as aliquots at -80°C until the day of vaccination (21). For *in vitro* validation studies, the mouse macrophage cell line J774A.1 cultured in DMEM supplemented with 10% FBS was infected at a 10:1 multiplicity of infection with control or GUCY2C Lm. After a 1 h incubation at 37°C, cells were washed 2x in PBS, resuspended in media containing 10 ug/mL gentamicin to eliminate free extracellular bacteria, and incubated an additional 5 h at 37°C. For immunofluorescence studies, Lm was labeled prior to infection by incubating with 2 mM CellTracker Red CMPTPX dye for 10 min at 37°C, and GUCY2C protein was stained using the anti-GUCY2C monoclonal antibody MS20 (22) followed by incubation with a peroxidase-conjugated 2° antibody for subsequent tyramide-FITC amplification. For western blot studies, protein was extracted from cells using M-PER reagent (Pierce) supplemented with protease inhibitors. GUCY2C protein was stained using MS20 (22) and p60 was stained using the anti-p60 monoclonal antibody p6017 (AdipoGen).

Replication-deficient adenovirus serotype 5 (Ad5) expressing mouse GUCY2C_1-429_ fused to the influenza HA_107-119_ CD4^+^ T-cell epitope known as S1 (Ad5-GUCY2C) was used as a positive control for generating GUCY2C-specific CD8^+^ T-cell responses (23). The adenoviral vaccine used in this study was produced by the Baylor College of Medicine in the Cell and Gene Therapy Vector Development Lab and certified to be negative for replication-competent adenovirus, mycoplasma, and host cell DNA contamination. All peptides used for experiments were custom synthesized by ThermoFisher Scientific and purified by HPLC to >95% purity.

### Mice and Immunizations

Studies employed BALB/cJ mice (Jackson Laboratories). For Lm vaccinations, Lm aliquots were thawed on the day of use, incubated at 37°C for 60 min in BHI broth, washed 2x in PBS, and resuspended to a concentration of 5x10^7^ colony-forming units (CFU)/mL in PBS. Mice were immunized intraperitoneally (i.p.) with 10^7^ CFU of recombinant Lm vaccine. For Ad5 vaccinations, mice were immunized intramuscularly (i.m.) with 10^10^ vp of Ad5-GUCY2C delivered as two 50 uL injections, one in each hind limb. All studies employed a single administration of Lm or Ad5 priming followed by Lm boosting (prime-boost studies indicated in **Figure 2**). For priming experiments, mice were sacrificed 7 d following Lm vaccination. For prime-boost immunizations, Ad5 and Lm vaccines were administered 21 d apart, and animals were euthanized 6 d after final vaccination.

### IFNγ ELISpot Assay

Enzyme-Linked Immunospot Assay (ELISpot) was performed using a mouse interferon-γ (IFNγ) single color ELISpot kit (Cellular Technology Limited) according to the manufacturer’s protocol. The evening before an experiment, 96-well plates were coated and incubated overnight at 4°C with IFNγ capture antibody. After splenocytes were isolated from immunized mice on the following day, plates were washed with PBS. Splenocytes were then plated in triplicate in a 0.1% DMSO solution of CTL-TEST medium (Cellular Technology Limited) with 10 ug/mL of peptide and incubated at 37°C for 24 h. For TCR avidity studies, splenocytes were pulsed with decreasing concentrations of GUCY2C_254-262_ peptide (10 ug/mL to 3 pg/mL) (23–25). The next day, splenocytes were removed, and development reagents were added to detect IFNγ-producing spot-forming cells (SFCs). The number of SFCs/well was determined using the SmartCount and Autogate functions of an ImmunoSpot S6 Universal Analyzer (Cellular Technology Limited). Peptide-specific responses were calculated by subtracting mean spot counts of 0.1% DMSO wells from peptide-pulsed wells.

### MHC Class I Stability Assay

The TAP-deficient cell line RMA-S expressing the MHC class I molecule H-2K^d^ (26) was kindly provided by Dr. Sean Murphy (University of Washington) and was used for peptide-MHC stability experiments. As previously described (27), RMA-S-H-2K^d^ cells were incubated overnight at 26°C with 30 ug/mL of each peptide. In the morning, cells were incubated 2 h at 37°C, washed 3x with PBS to remove unbound peptide, and incubated an additional 2, 4, or 6 h at 37°C. Cells were then stained with anti-H-2K^d^-PE antibody (Invitrogen, Clone SF1-1.1.1), and surface H-2K^d^ was quantified as mean fluorescence intensity (MFI) by flow cytometry. The percent change in surface peptide-H-2K^d^ complexes was calculated using the formula, $\frac{MFI^{peptide}-MFI^{no\;peptide}}{MFI^{0\;hrs}-MFI^{no\;peptide}}\times 100$ (28). The t_1/2_ for the peptide-MHC complex was calculated by nonlinear regression.

### Tumor Studies

The murine CT26 colorectal cancer cell line expressing mouse GUCY2C and luciferase (23) was used for *in vivo* tumor studies. Seven days after final immunizations, mice received 5x10^5^ CT26 cells *via* i.v. tail vein injection to model metastatic colorectal cancer recurrence in the lungs. Tumor burden was quantified by subcutaneous injection of 3.75 mg of D-luciferin potassium salt (Gold Biotechnologies) in PBS solution. Following an 8 min incubation, mice were imaged with a ten-second exposure using a Caliper IVIS Lumina XR imaging station (PerkinElmer). Total radiance (photons/second) was quantified by Living Image *In Vivo* Imaging Software (PerkinElmer).

## Results

### GUCY2C_254-262_ Is Subdominant to Lm-Derived Epitopes

**Figure 1:**
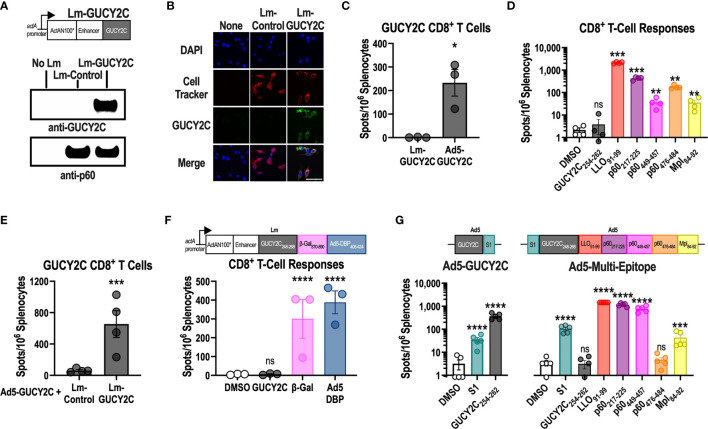
GUCY2C_254-262_ peptide is subdominant to Lm-derived peptides. **(A)** Lm-GUCY2C secretes a fusion protein comprised of ActAN100*, an enhancer sequence, and mouse GUCY2C_23-429_ under control of the *actA* promoter. **(A, B)** J774A.1 macrophages were uninfected or infected with Lm-Control or Lm-GUCY2C at a 10:1 MOI for 6 h at 37°C. GUCY2C fusion protein was detected by **(A)** western blot and **(B)** immunofluorescence. Scale bars in B are 50 um. **(C, D)** BALB/c mice (n=3-4/group) were immunized intraperitoneally (i.p.) with 10^7^ colony-forming units (CFU) of recombinant Lm secreting GUCY2C (Lm-GUCY2C) or intramuscularly (i.m.) with 10^10^ vp of Ad5-GUCY2C. **(E)** Animals received 10^7^ CFU of Lm-GUCY2C or Lm-Control 21 d after priming with 10^10^ vp of Ad5-GUCY2C. **(C–E)** Fourteen days after Ad5-GUCY2C immunization or 6-7 d after Lm administrations, mice were euthanized, and splenocytes were collected to quantify GUCY2C_254-262_ **(C, E)** or Lm-specific **(D)** T-cell responses, quantified by IFNγ ELISpot. **(F)** BALB/c mice (n=3) were immunized with an Lm containing a multi-epitope construct composed of GUCY2C, β-Gal, and Ad5 DBP epitopes (as depicted), and those responses were quantified 7 d later by IFNγ ELISpot. **(G)** BALB/c mice (n=5/group) were immunized with Ad5-GUCY2C (left) or a recombinant Ad5 containing S1, GUCY2C_254-262_, and the H-2K^d^-restricted Lm epitopes as depicted (right). Responses were quantified 14 d later by IFNγ ELISpot. Statistical comparisons were made by unpaired T test **(C, E)** or one-way ANOVA with Bonferroni correction for multiple comparisons of epitope-specific response to control DMSO wells **(D**, **F**, **G)**. Error bars indicate mean +/- SEM. Symbols indicate individual animals. *P < 0.05; **P < 0.01; ***P < 0.001; ****P < 0.0001; ns, not significant.

Lm-GUCY2C was produced using a construct composed of the *actA* promoter, an enhancer, and a fusion protein of a modified version of the first 100 amino acids of actA (ActAN100*) and residues 23-429 of murine GUCY2C (**Figure 1A** and **Supplementary Figure 1**). Lm-GUCY2C successfully produces GUCY2C protein upon infection of J744A.1 macrophages, detected in the supernatant (**Figure 1A**) and in the cells (**Figure 1B**). However, while Ad5-GUCY2C induced robust GUCY2C-specific CD8^+^ T-cell responses, Lm-GUCY2C vaccination failed to prime CD8^+^ T cell responses to GUCY2C (**Figure 1C**). In contrast, Lm-GUCY2C induced robust Lm-specific CD8^+^ T-cell responses against multiple H-2K^d^-restricted Lm epitopes derived from Listeriolysin-O (LLO), p60, and metalloprotease (Mpl) antigens (**Figure 1D**). Moreover, Lm-GUCY2C successfully boosted GUCY2C-specific CD8+ T-cell responses that were primed 21 days earlier with Ad5-GUCY2C (**Figure 1E**). Furthermore, in the context of a multi-epitope Lm vaccine that included epitopes from GUCY2C, β-galactosidase, and Ad5, robust responses were produced against the highly immunogenic foreign antigens β-galactosidase and Ad5, but not the self-antigen GUCY2C (**Figure 1F**). Collectively, these data suggest that the only GUCY2C CD8^+^ T-cell epitope in BALB/c mice (GUCY2C_254-262_) (29) is processed and presented upon Lm-GUCY2C administration but is unable to prime naive GUCY2C_254-262_-specific CD8^+^ T-cell responses. In the context of primarily T cells with weak TCR-peptide-MHC interactions escaping self-tolerance (30, 31), we hypothesized that the GUCY2C_254-262_ epitope might be subdominant in the context of live Lm vaccine vectors. To confirm that Lm epitopes limit GUCY2C_254-262_ immunogenicity, we utilized the Ad5-GUCY2C vaccine template, which produces robust GUCY2C_254-262_ responses (29, 32–34), and created an Ad5 vaccine composed of GUCY2C_254-262_, the S1 CD4^+^ helper T-cell epitope used in Ad5-GUCY2C vaccines (35), and immunogenic epitopes from LLO, p60 and Mpl (**Figure 1G**). Strikingly, this construct induced robust responses directed to the S1 CD4^+^ T-cell epitope and Lm-derived CD8^+^ T-cell epitopes but failed to induce responses to GUCY2C_254-262_ (**Figure 1G**).

### Enhancing GUCY2C_254-262_ Quantity, Degradation, or Processing Does Not Overcome Immunodominance

**Figure 2:**
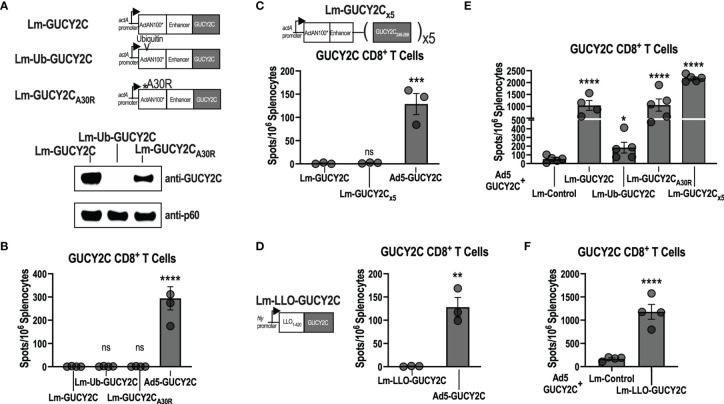
Lm-GUCY2C vaccines designed to enhance antigen processing do not improve GUCY2C_254-262_ immunogenicity. **(A)** Lm-GUCY2C used in **Figure 1** secretes a fusion protein consisting of ActAN100* combined with an enhancer sequence and GUCY2C. Lm-GUCY2C_A30R_ contains a point mutation at the N-terminal residue following the signal sequence of actA, and Lm-Ub-GUCY2C contains ubiquitin protein following the signal sequence within ActAN100* to enhance degradation, confirmed by western blot analysis. **(B)** BALB/c mice (n=3-4/group) were immunized i.p. with 10^7^ CFU of Lm vaccines or 10^10^ vp of Ad5-GUCY2C vaccine and euthanized 7 or 14 d later, respectively, to quantify GUCY2C-specific CD8^+^ T-cell responses by IFNγ ELISpot. **(C)** To enhance the quantity of GUCY2C_254-262_ peptide and improve antigen processing, Lm-GUCY2C_x5_ secretes a fusion protein containing five repeating copies of GUCY2C_248-268_ separated by sequences predicted to facilitate proteasomal cleavage. BALB/c mice (n=3-4/group) were immunized i.p. with 10^7^ CFU of Lm-GUCY2C_x5_ vaccine or 10^10^ vp of Ad5-GUCY2C vaccine and euthanized 7 or 14 d later, respectively, to quantify GUCY2C-specific CD8^+^ T-cell responses by IFNγ ELISpot. **(D)** Lm-LLO-GUCY2C secretes a fusion protein comprised of a truncated LLO protein fused to GUCY2C under control of the *hly* promoter. BALB/c mice (n=3/group) were immunized i.p with 10^7^ CFU of Lm-LLO-GUCY2C or i.m. with 10^10^ vp of Ad5-GUCY2C and GUCY2C-specific CD8^+^ T-cell responses were quantified by IFNγ ELISpot 7 or 14 d later, respectively. **(E, F)** BALB/c mice (n=4-5/group) were immunized i.m. with 10^10^ vp of Ad5-GUCY2C vaccine on day 0 followed by 10^7^ CFU of Lm vaccines i.p. on day 21, including Lm constructs designed to enhance epitope presentation employed in **(A–C)** and the LLO-based construct employed in **(D)**. Six days after Lm boosting, GUCY2C-specific CD8^+^ T-cell responses were quantified by IFNγ ELISpot. Statistical comparisons were made to Lm-GUCY2C using one-way ANOVA with Bonferroni correction for multiple comparisons **(B, C, E)** or unpaired T test **(D, F)**. Error bars indicate mean +/- SEM. Symbols indicate individual animals. *P < 0.05; **P < 0.01; ***P < 0.001; ****P < 0.0001; ns, not significant.

During microbial infections, antigens are degraded into short peptide fragments and loaded onto MHC molecules for presentation to T cells (36). Interestingly, while a single bacterium expresses thousands of potentially antigenic proteins (37), the immune system concentrates T-cell responses towards only a few “dominant” antigenic sequences. In contrast, sequences that induce T-cell responses to a lesser, or undetectable, degree are termed “subdominant.” Because GUCY2C_254-262_ is subdominant to Lm epitopes in Lm-GUCY2C, we hypothesized that it might be possible to rescue Lm-GUCY2C immunogenicity by increasing the relative quantity of GUCY2C epitopes and produced a variety of Lm vaccines expressing GUCY2C in different contexts to test that hypothesis (**Figure 2A**). It has been reported that increasing the degradation rate of an antigen can enhance its immunogenicity (38–40). Thus, we mutated the N-terminal residue following the actA signal sequence (A30R) to destabilize the actA-GUCY2C fusion protein or incorporated ubiquitin into our actA-GUCY2C fusion protein (Ub-GUCY2C) to promote its degradation (41). We then cloned these constructs into Lm, generating the vaccines Lm-GUCY2C_A30R_ and Lm-Ub-GUCY2C, respectively (**Figure 2A**). While both constructs resulted in increased degradation and much lower steady-state levels of GUCY2C (**Figure 2A**), vaccination with these constructs did not improve GUCY2C_254-262_ immunogenicity (**Figure 2B**). Recently, a group developing an Lm vaccine against the EGFRvIII variant reported that EGFRvIII immunogenicity was enhanced when repeating copies of epitope were encoded and flanked by sequences predicted to facilitate proteasomal cleavage (42). To similarly increase the copy number and improve processing of GUCY2C_254-262_ peptide, we used a similar design with 5 copies flanked by sequences designed to facilitate antigen processing (Lm-GUCY2C_x5_; **Figure 2C**). However, Lm-GUCY2C_x5_ similarly failed to induce GUCY2C_254-262_-specific CD8^+^ T-cell responses (**Figure 2C**). Finally, we altered the promoter and fusion protein by which GUCY2C is expressed. Lm vaccines advanced into clinical trials have predominantly expressed vaccine antigen under the *hly* promoter fused to LLO or under the *actA* promoter fused to actA (13). Importantly, antigen expression kinetics during infection also influences antigen immunogenicity (43), and *hly* and *actA* promoters are distinctly regulated (44). Moreover, fusion to LLO also has been reported to enhance subdominant epitope immunogenicity in some cases (45). Thus, we fused GUCY2C to LLO and expressed it under the *hly* promoter (Lm-LLO-GUCY2C; **Figure 2D**); however, Lm-LLO-GUCY2C also failed to prime GUCY2C-specific CD8^+^ T-cell responses (**Figure 2D**). Notably, while these Lm-GUCY2C variants were unable to prime responses, all were capable of boosting GUCY2C-specific memory CD8^+^ T-cell responses in mice primed with Ad5-GUCY2C (**Figure 2E, F**). Finally, recent studies report that subdominant T-cell clones express high levels of PD-1 upon activation, resulting in cell death, and that administration of PD-1-blocking antibody during vaccination significantly improves the expansion of subdominant T-cell clones (46, 47). However, treatment with anti-PD-1 antibody during Lm-GUCY2C immunization did not produce GUCY2C-specific CD8^+^ T-cell priming (**Supplementary Figure 2**).

### Lm-Derived Epitopes Exhibit Superior Peptide-MHC Stability Compared to GUCY2C_254-262_

**Figure 3:**
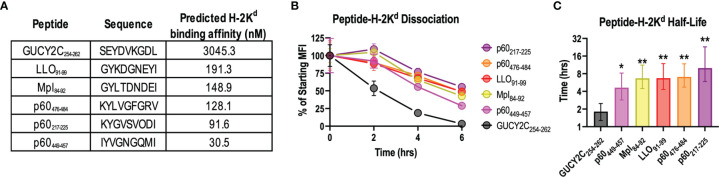
The GUCY2C_254-262_-H-2K^d^ complex is less stable than peptide-H-2K^d^ complexes derived from Lm epitopes. **(A)** The predicted binding affinity of GUCY2C_254-262_ peptide for H-2K^d^ was compared to that of all known Lm-specific H-2K^d^-restricted epitopes using NetMHCPan4.0. **(B, C)** The stability of peptide-H-2K^d^ complexes was measured using the TAP-deficient cell line, RMA-S H-2K^d^. **(B)** The normalized specific mean fluorescence intensities (MFI) are shown. Error bars indicate mean +/- SEM of technical replicates. **(C)** Half-lives (t_1/2_) were determined by nonlinear regression of **(B)**. Error bars indicate computed t_1/2_ +/- 95% confidence intervals. Statistical comparisons were made to GUCY2C_254-262_ by extra sum-of-squares F Test with Bonferroni correction. Data are representative of two experiments. *P < 0.05; **P < 0.01.

In addition to antigen quantity (48), temporal expression (43), and degradation (49), competition between immunodominant and subdominant epitopes may reflect differing affinities for MHC class I and/or stabilities of the peptide-MHC complex (50). Thus, we compared the predicted binding affinity of GUCY2C_254-262_ for its cognate MHC class I molecule, H-2K^d^, with that of all known H-2K^d^-restricted Lm epitopes using NetMHCPan4.0 (**Figure 3A**). Notably, the predicted binding affinity of GUCY2C_254-262_ for H-2K^d^ was more than 15x lower than that of all Lm-derived peptides (**Figure 3A**). We next compared the stability of the GUCY2C_254-262_-H-2K^d^ complex to that of the Lm epitopes using the TAP-deficient cell line, RMA-S, stably expressing H-2K^d^. In the absence of TAP, cytosolic peptides are not transported to the endoplasmic reticulum, resulting in empty MHC molecules that are highly unstable and rapidly internalized from the cell surface (51). However, the addition of exogenous peptide stabilizes MHC molecules on the cell surface, and the stability of different peptide-MHC complexes can be compared by the decay of the complex and reduced surface MHC levels (52). Thus, to assess the stability of the GUCY2C and Lm peptide-MHC complexes, we quantified the decay of surface peptide-H-2K^d^ over time (**Figure 3B**). As predicted from the *in silico* affinity algorithms (**Figure 3A**), GUCY2C_254-262_-H-2K^d^ complexes rapidly decayed from the cell surface compared to complexes formed with Lm epitopes (**Figure 3B**). Nonlinear regression analyses estimate the t_1/2_ for GUCYC_254-262_-H-2K^d^ complexes at 1.8 h, while the t_1/2_ for H-2K^d^ complexes with Lm epitopes ranged from 4.6 to 10.0 h (**Figure 3C**). Collectively, these data suggest that the GUCY2C_254-262_ epitope forms an unstable complex with its cognate MHC class I molecule, H-2K^d^, compared to H-2K^d^ complexes formed with dominant Lm epitopes.

### F255Y Mutation of GUCY2C_254-262_ Enhances Stability of the GUCY2C_254-262_-H-2K^d^ Complex

**Figure 4:**
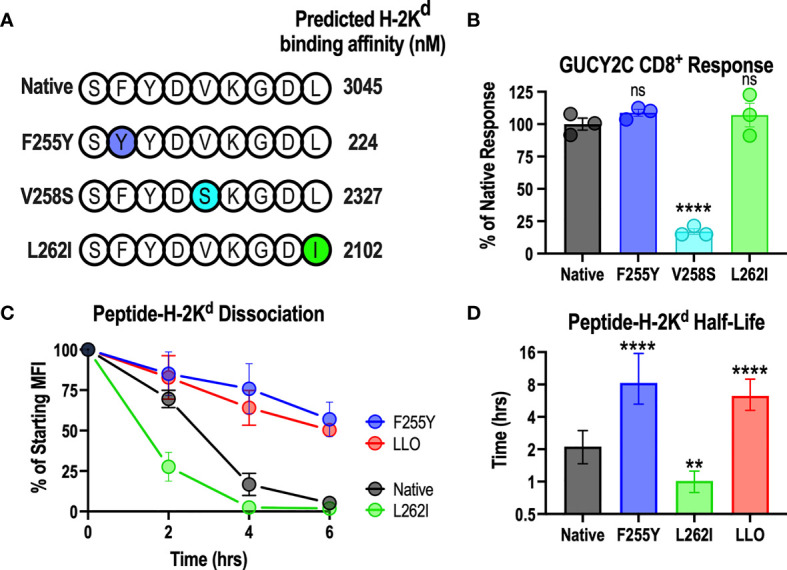
Anchoring residue modification improves the stability of the GUCY2C_254-262_-H-2K^d^ complex. **(A)** Shows the amino acid sequence of native and altered peptides predicted with NetMHCPan 4.0 to improve H-2K^d^ binding affinity. **(B)** Splenocytes from mice immunized against the native GUCY2C_254-262_ epitope with Ad5-GUCY2C were incubated overnight with native GUCY2C_254-262_ peptide or altered GUCY2C peptides, and the number of IFNγ-producing T cells were quantified by ELISpot (relative to recognition of the native GUCY2C_254-262_ peptide). Normalized spot counts were compared by one-way ANOVA to native GUCY2C_254-262_ peptide with Bonferroni correction for multiple comparisons. Error bars indicate mean +/- SEM of technical replicates. **(C)** The stability of the peptide-H-2K^d^ complexes was assessed using RMA-S H-K^d^ cells, and the normalized specific MFIs are shown. Error bars indicate mean +/- SEM of two experiments. **(D)** Half-lives (t_1/2_) were determined by nonlinear regression of **(C)**. Error bars indicate computed t_1/2_ +/- 95% confidence intervals. Statistical comparisons were made to native GUCY2C by extra sum-of-squares F Test with Bonferroni correction. **P < 0.01; ****P < 0.0001; ns, not significant.

The above data demonstrate that the GUCY2C_254-262_ epitope interacts weakly with H-2K^d^ compared to dominant Lm epitopes in the Lm-GUCY2C vaccine (**Figure 3**). Notably, the strength of the peptide-MHC interaction is most significantly impacted by a few amino acids at specific locations along the peptide (anchoring residues). At anchoring residues, amino acids from the peptide are partially or fully buried within the pockets of the MHC molecule, forcing significant peptide-MHC interactions at these locations (53). As a result, amino acids at these positions are highly conserved for each MHC allele. For the H-2K^d^ allele, anchoring residues are located at positions 2, 5, and 9 along the peptide (53). Employing the most conserved amino acid at each anchoring residue position for immunogenic H-2K^d^ epitopes (53), we generated three different GUCY2C_254-262_ peptide variants, each using the preferred amino acid at a given anchor, resulting in improved predicted affinity for H-2K^d^ (**Figure 4A**). To confirm that amino acid substitution at anchoring residues did not alter recognition by T-cells specific for the native GUCY2C_254-262_ epitope, splenocytes from mice primed against the native sequence (Ad5-GUCY2C) were employed in an ELISpot assay with native GUCY2C_254-262_, GUCY2C_F255Y_, GUCY2C_V258S_, or GUCY2C_L262I_ point mutations (**Figure 4B**). Notably, GUCY2C_V258S_ was poorly recognized by GUCY2C_254-262_-specific T-cells (**Figure 4B**) and was not explored further. However, GUCY2C_F255Y_ and GUCY2C_L262I_ peptide recognition by GUCY2C_254-262_-specific CD8^+^ T-cells was equivalent to recognition of the native GUCY2C_254-262_ peptide sequence (**Figure 4B**). Next, we assessed the stability of the GUCY2C_254-262_-H-2K^d^ complex between native, GUCY2C_F255Y_, and GUCY2C_L262I_ sequences (**Figures 4C, D**). As expected, the GUCY2C_F255Y_-H-2K^d^ complex was significantly more stable than the native GUCY2C complex and shifted the half-life from 2.1 h to 8.3 h, respectively. However, the GUCY2C_L262I_-H-2K^d^ complex was less stable (1.0 h) than the native sequence. Thus, the GUCY2C_F255Y_ peptide is recognized similarly to the native peptide sequence by T cells primed against native GUCY2C (**Figure 4B**) but forms significantly more stable complexes with H-2K^d^ (**Figure 4C, D**).

### GUCY2C_F255Y_ Mutation Rescues Lm-GUCY2C Immunogenicity and Antitumor Efficacy

**Figure 5:**
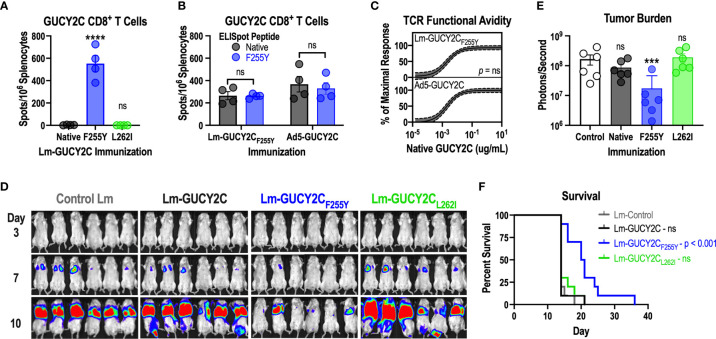
Lm-GUCY2C_F255Y_ primes GUCY2C-specific CD8^+^ T-cell responses and antitumor immunity. **(A)** BALB/c mice (n=4/group) were immunized i.p. with 10^7^ CFU of Lm secreting native GUCY2C or GUCY2C with altered GUCY2C_254-262_ epitopes, and GUCY2C_254-262_-specific T-cell responses were quantified 7 d later by IFNγ ELISpot. **(B, C)** BALB/c mice (n=4/group) were immunized i.p. with 10^7^ CFU of Lm-GUCY2C_F255Y_ or i.m. with 10^10^ vp of Ad5-GUCY2C and euthanized 7 or 14 d later, respectively. **(B)** Splenocytes from immunized mice were pulsed with native GUCY2C_254-262_ peptide or GUCY2C_F255Y_ peptide to assess the magnitude of responses by IFNγ ELISpot. **(C)** Splenocytes from immunized mice were pulsed with decreasing concentrations of native GUCY2C_254-262_ peptide to assess functional TCR avidity. Nonlinear regression (solid line) of GUCY2C-specific CD8^+^ T-cell avidity is depicted with 95% confidence intervals (dashed lines). Statistical comparison was made by extra sum-of-squares F Test. **(D–F)** BALB/c mice (n=10/group) were immunized i.p. with 10^7^ CFU control, GUCY2C, GUCY2C_L262I_, or GUCY2C_F255Y_ Lm. Seven days later, mice were challenged with the CT26 colorectal cancer cell line expressing GUCY2C and luciferase. **(D)** Tumor burden was monitored by bioluminescence imaging and quantified on day 7 **(E)**. **(F)** Survival was monitored longitudinally. Data were analyzed by one-way **(A, E)** or two-way **(B)** ANOVA with Bonferroni correction for multiple comparisons to compare 1 or 2 variables, respectively. Error bars indicate mean +/- SEM. Survival **(F)** was analyzed by the Mantel-Cox log rank test with all immunized groups compared to control with Bonferroni correction. Symbols indicate individual animals. ***P < 0.001; ****P < 0.0001; ns, not significant.

Having demonstrated that GUCY2C_254-262_ peptide interacts weakly with H-2K^d^ compared to peptides from Lm (**Figure 3**) and that GUCY2C_F255Y_ modification significantly improves the stability of the peptide-MHC complex (**Figure 4**), we hypothesized that improved stability of the GUCY2C_F255Y_ peptide with H-2K^d^ may elevate GUCY2C_254-262_ within the Lm immunodominance hierarchy. Thus, we cloned new Lm vaccines identical to Lm-GUCY2C but containing the GUCY2C_F255Y_ (Lm-GUCY2C_F255Y_) or GUCY2C_L262I_ (Lm-GUCY2C_L262I_) modifications and assessed their ability to induce responses recognizing the native GUCY2C_254-262_ epitope by IFNy ELISpot (**Figure 5A**). Indeed, improving the stability of the GUCY2C_254-262_-H-2K^d^ complex with GUCY2C_F255Y_ (**Figure 4**) rescued GUCY2C immunogenicity following Lm-GUCY2C_F255Y_ vaccination (**Figure 5A**). Similarly, Lm-GUCY2C_L262I_ failed to rescue responses (**Figure 5A**), reflecting worsened stability (**Figure 4**). Given that altering epitope residues may impact T-cell recognition (54), we next examined cross-reactivity of responses elicited to native and mutated GUCY2C_F255Y_, as well as the avidity of the vaccine-induced T-cell pools for native GUCY2C_254-262_. Notably, T-cells from mice immunized with native Ad5-GUCY2C or Lm-GUCY2C_F255Y_ were equally cross-reactive to native and GUCY2C_F255Y_ peptides (**Figure 5B**). Moreover, the avidity of T-cell pools induced by Ad5-GUCY2C and Lm-GUCY2C_F255Y_ immunization were identical for native GUCY2C_254-262_ epitope (**Figure 5C**). Together, these data suggest that the GUCY2C_254-262_-reactive CD8^+^ T-cell pool is similar in mice primed against the native GUCY2C and GUCY2C_F255Y_ sequences. Finally, we confirmed that Lm-GUCY2C_F255Y_ induces antitumor immunity against colorectal cancer cells expressing native GUCY2C (**Figures 5D–F**). Mice were immunized with Lm-Control, Lm-GUCY2C, Lm-GUCY2C_L262I_, or Lm-GUCY2C_F255Y_ in a model of recurrent, metastatic colorectal cancer using the CT26 cell line expressing GUCY2C and firefly luciferase. As expected, the priming of GUCY2C_254-262_-specific CD8^+^ T cells by Lm-GUCY2C_F255Y_ reduced metastatic tumor burden (**Figures 5D, E**) and improved survival (**Figure 5F**), in striking contrast to Lm-GUCY2C and Lm-GUCY2C_L262I_, reflecting the immunodominance of Lm epitopes in those vaccines.

## Discussion

The broader vaccine field, as well as cancer vaccines specifically, employs a wide variety of vaccination platforms. Indeed, a recent review assessing clinical trials testing personalized cancer vaccines between 2017 and 2020 found that methods of cancer vaccination are highly variable and noted that a “…lack of consensus on the best approach to the systematic testing of antigen and vaccine efficacy was evident” (8). Of 23 trials analyzed, 6 utilized mRNA, 6 utilized peptide, 5 utilized *Listeria monocytogenes*, 3 utilized DNA, 2 utilized mRNA in combination with a viral vector, and 1 utilized yeast. These observations underscore the necessity to evaluate current methods to define optimal methods of cancer vaccination.

In that context, studies here examining a GUCY2C-directed Lm vaccine reveal a significant limitation of this vaccine platform. Notably, while Lm-GUCY2C induces robust Lm-specific CD8^+^ T-cell responses, it cannot prime GUCY2C-specific CD8^+^ T-cell responses (**Figure 1B**). In contrast, Lm-GUCY2C generates potent secondary expansion of GUCY2C-specific memory CD8^+^ T-cells (**Figure 1D**), suggesting that the primary GUCY2C_254-262_ epitope is immunologically active and presented by MHC but is subdominant to Lm epitopes, depending on the context (primary versus secondary GUCY2C exposure). Alternatively, this may reflect low abundance of GUCY2C_254-262_ epitope, falling below levels needed for priming, but above levels needed for boosting. The lack of priming with Lm-GUCY2C variants that enhance epitope abundance (**Figure 2**) suggest that abundance alone doesn’t explain its failure to prime. Instead, this appears to reflect the poor stability of the GUCY2C_254-262_-H-2K^d^ complex compared to Lm-derived peptide-H-2K^d^ complexes (>5-fold faster dissociation from MHC; **Figure 3**), which can be reversed by an altered peptide-ligand with enhanced peptide-MHC stability (**Figure 4**), Lm-GUCY2C_F255Y_, rescuing immunogenicity and antitumor efficacy (**Figure 5**). Collectively, these findings suggest that Lm-derived peptides may act as a significant competitive barrier to target antigen presentation and immunogenicity, limiting the effectiveness of Lm-based vaccines.

In the context of Lm-based cancer vaccines, there are multiple reports of limited immunogenicity when using Lm vaccines to prime CD8^+^ T-cell responses against cancer antigens. For example, Lm vaccines against the tumor-associated antigens PAP (55) and mesothelin (56) had poor immunogenicity alone but were more effective as booster vaccines in the context of a heterologous prime-boost immunization regimen, similar to observations with GUCY2C (**Figure 1**) (57). However, Lm vaccines alone against HER2 (58) and PSA (59) tumor antigens induce robust antitumor immunity. Interestingly, those studies employed foreign antigens (human HER2 or human PSA) in mice, which are not limited by self-tolerance mechanisms. Moreover, Lm-based immunization with HER2 in rat HER2 transgenic mice (60) or immunization with HPV-16 E7 in HPV16 E6/E7 transgenic mice (61) did successfully induce responses, despite limitations imposed by self-tolerance. Collectively, these studies suggest that the immunogenicity of Lm vaccines is likely dependent on the vaccine antigen and context and that weak antigens (some self-antigens like GUCY2C and some foreign antigens) may be more susceptible to competition with Lm-derived peptides than other antigens. Notably, a recent clinical trial testing an Lm-derived vaccine against mesothelin reported that T-cell responses towards mesothelin were inconsistent and lower in magnitude than LLO (14). Similarly, Lm expressing 4 prostate cancer antigens induced significantly lower and inconsistent responses to the prostate antigens than LLO in patients, resulting in early study termination (62). Together, these observations suggest that competition between immunodominant Lm antigens and vaccine antigens is conserved in humans.

Interestingly, Ad5-based vaccines expressing GUCY2C are highly capable of priming GUCY2C-specific immunity, despite containing viral peptides that could theoretically compete with GUCY2C epitopes for presentation. However, competition between Ad5 and GUCY2C peptides is likely significantly less than the competition between Lm and GUCY2C peptides for at least two reasons. First, the Ad5 genome contains roughly 40 protein-coding genes (63), while the Lm genome encompasses over 2800 proteins (64), creating significantly more opportunities for immunodominance over GUCY2C. Second, despite being attenuated to limit inter-cellular migration and pathogenicity, Lm vaccines are replication-competent. Thus, the synthesis and presentation of GUCY2C antigen is concurrent with the production and presentation of Lm antigens in infected APCs. In contrast, Ad5 vectors utilized clinically and in the present studies are replication-deficient, and target antigens are encoded within the episomal Ad5 genomic DNA. Thus, during the course of infection, the quantity of Ad5 epitope presentation is predicted to peak at injection and diminish over time as the administered vector is cleared, while GUCY2C presentation is initially absent until it is *de novo* synthesized by the host cell over a much more extended period. The lag time between Ad5 exposure and the synthesis of target antigen by the host cell may, therefore, allow for temporal separation between presentation of GUCY2C and Ad5 epitopes – a working hypothesis supported by previous studies revealing the ability of GUCY2C-conjugated, but not Ad5 structural, antigens to provide CD4^+^ T-cell help to GUCY2C-specific CD8^+^ T cells (35). Thus, a surplus of Lm antigens, in addition to a lack of temporal separation between Lm and target antigen presentation, may operate to restrict vaccine-directed immune responses in the context of Lm vaccines.

This is the first study to demonstrate that Lm-derived peptides may interfere with the immunogenicity of target vaccine antigens. These studies carry significant implications for designing and implementing Lm-based cancer vaccines. Specifically, Lm vaccines may be inferior at priming vaccine-specific responses than other vaccine platforms. While use of altered peptide ligands with enhanced peptide-MHC stability could ameliorate this drawback, the identification and validation of altered peptide ligands for relevant patient HLA alleles is a cumbersome endeavor. Therefore, using other vaccine vectors for priming immune responses may be more practical. Alternatively, Lm engineering approaches, such as episomal plasmid-based transgene expression (45, 58, 59, 65) may produce higher antigen expression than the approach used here, in which a single chromosomal copy of GUCY2C is created (12). Despite the limitation identified here, Lm vaccines may be particularly useful in a different context. Specifically, multiple studies have demonstrated the ability of Lm vaccines to boost memory CD8^+^ T-cell responses (55, 56). Additionally, preclinical and clinical studies suggest that Lm vaccines transform tumor microenvironments by increasing CD8^+^ T-cell infiltration (66), decreasing immunosuppressive regulatory T cells (10, 65) and myeloid-derived suppressor cells (11, 65), and repolarizing tumor-associated macrophages from M2 to M1 phenotypes (10, 66, 67). While the capabilities of Lm to boost CD8^+^ T-cell responses and remodel immunosuppressive tumor microenvironments may be a unique advantage compared to other platforms, the clinical significance of these findings and the extent to which other vaccine platforms compare has yet to be fully investigated. Moreover, any superiorities of Lm-based vaccines must also be weighed against the potential risks and toxicities associated with using a live bacterial platform. Indeed, despite the numerous successes developing vaccines to elicit protective antibody responses, often with attenuated or inactivated pathogens, very few vaccines that elicit protective CD8⁺ T-cell responses have been clinically successful. Continued investigation of currently available vaccine vectors and development of novel vaccine approaches and combinations are necessary to elicit robust CD8⁺ T-cell responses against cancer and intractable pathogens to address this unmet need.

## Data Availability Statement

The raw data supporting the conclusions of this article will be made available by the authors, without undue reservation.

## Ethics Statement

The animal study was reviewed and approved by Thomas Jefferson University Institutional Animal Care and Use Committee (IACUC).

## Author Contributions

These investigations were conducted, supporting methodologies were developed, and data were acquired and validated by JF, JS, YY, RC, and JB. Data were visualized, and the original draft was prepared, by JF. The project was conceptualized, data were reviewed, and the manuscript was reviewed and edited by all authors. AS provided administration and acquisition of financial support for the project leading to this publication. All authors contributed to the article and approved the submitted version.

## Funding

This work was supported by grants to SW from NIH (1R01 CA204881, 1R01 CA206026), the Department of Defense Congressionally Directed Medical Research Program W81XWH-17-PRCRP-TTSA, The Courtney Ann Diacont Memorial Foundation, and Targeted Diagnostic & Therapeutics, Inc; and to AS from the Department of Defense Congressionally Directed Medical Research Program W81XWH-19-1-0263, W81XWH-19-1-0067, and W81XWH-17-1-0299, and David and Lorraine Swoyer. The funders were not involved in the study design, collection, analysis, interpretation of data, the writing of this article, or the decision to submit it for publication. JF was supported by the Alfred W. and Mignon Dubbs Fellowship Fund and a PhRMA Foundation Pre-Doctoral Fellowship in Pharmacology/Toxicology. RC was supported by the T32 Training Program in Cancer Biology (5T32 CA236736-02). JB was supported by an NIH Ruth Kirschstein Individual Predoctoral MD/PhD Fellowship (F30 DK127639). SW is the Samuel M.V. Hamilton Professor of Thomas Jefferson University. Research reported in this publication utilized the Flow Cytometry and Lab Animal Shared Resources of the Sidney Kimmel Cancer Center at Jefferson Health and was supported by the National Cancer Institute of the National Institutes of Health under Award Number 5P30 CA056036-20. The content is solely the responsibility of the authors and does not necessarily represent the official views of the NIH.

## Conflict of Interest

SW is a member of the Board and Chair of the Scientific Advisory Board of, and AS is a consultant for, Targeted Diagnostics & Therapeutics, Inc., which provided research funding that, in part, supported this work and has a license to commercialize inventions related to this work.

The remaining authors declare that the research was conducted in the absence of any commercial or financial relationships that could be construed as a potential conflict of interest.

## Publisher’s Note

All claims expressed in this article are solely those of the authors and do not necessarily represent those of their affiliated organizations, or those of the publisher, the editors and the reviewers. Any product that may be evaluated in this article, or claim that may be made by its manufacturer, is not guaranteed or endorsed by the publisher.
