# Supplementary material for: T-Cell Responses to Immunodominant Listeria Epitopes Limit Vaccine-Directed Responses to the Colorectal Cancer Antigen, Guanylyl Cyclase C
Source: Front Immunol. 2022 Mar 9;13:855759. doi: 10.3389/fimmu.2022.855759 (PMC8959893; doi:10.3389/fimmu.2022.855759)
Supplement: Supplementary file 1 [file DataSheet_1.pdf]

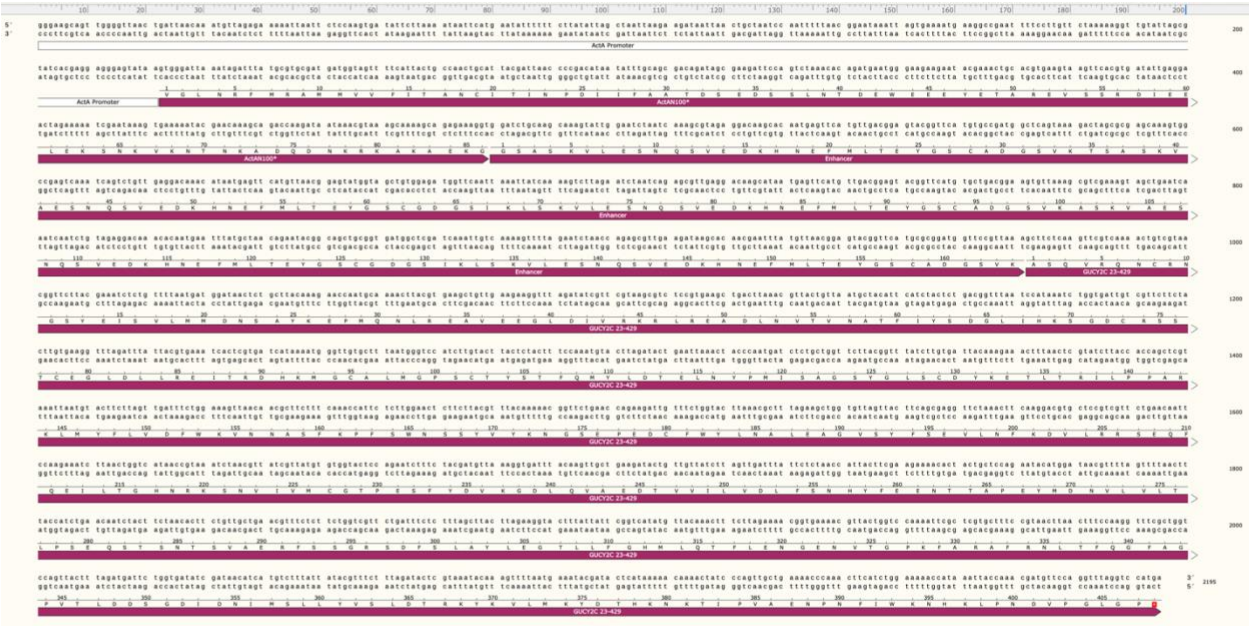

Supplementary Figure 1. Sequence of Lm-GUCY2C.

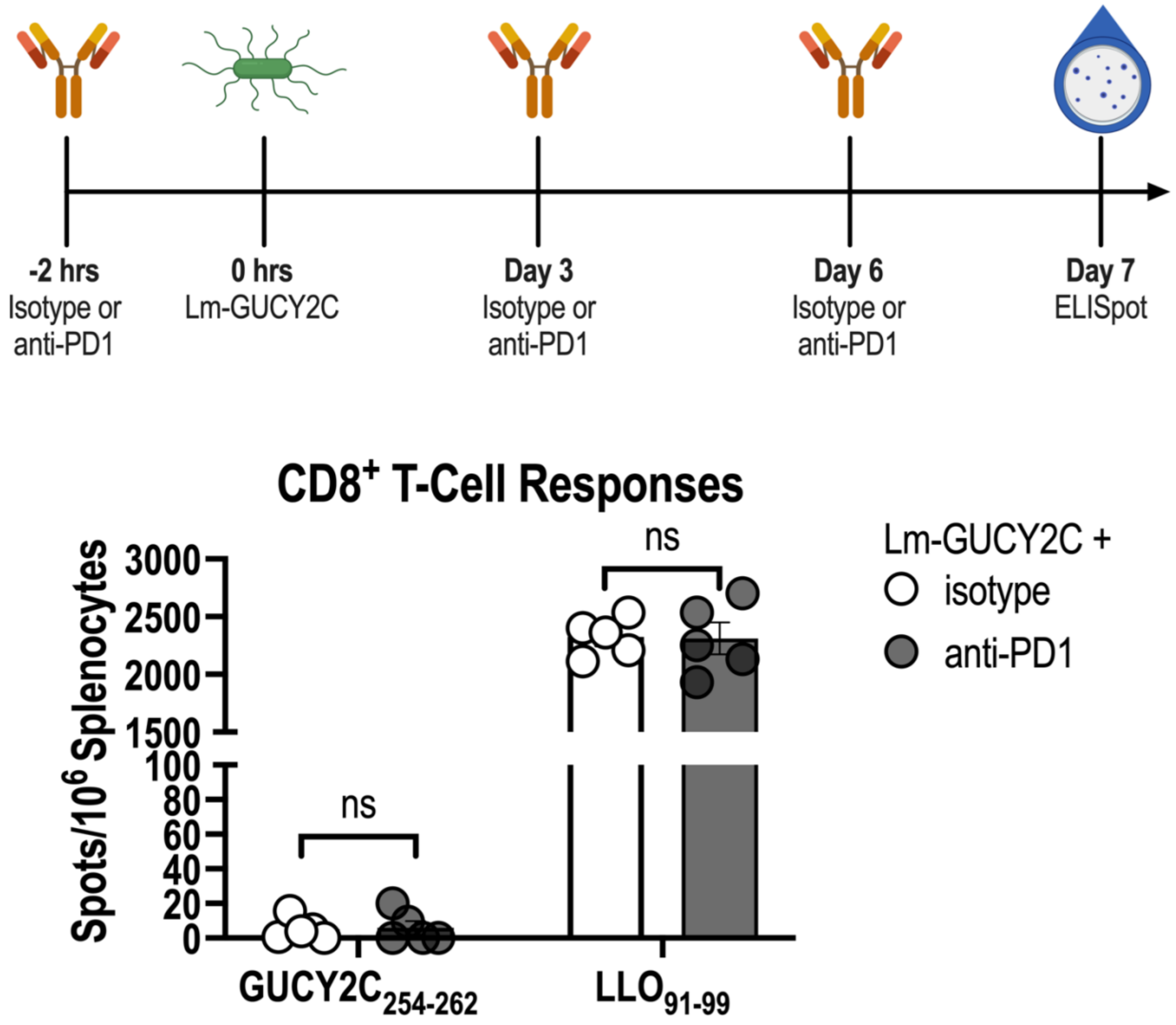

Supplemental Figure 2. PD1 blockade does not reverse subdominance. BALB/c mice (n=5/group) were treated i.p. with 100 ug of anti-PD1 antibody (InVivoMab, clone RMP1-14) or isotype control (InVivoMab clone 2A3) 2 h before i.p. vaccination with 10^7 CFU of Lm-GUCY2C. On days 3 and 6 after vaccination, mice received an additional 100 ug of antibody and on day 7 GUCY2C and LLO -specific $CD8^+$ T cells were quantified by IFNγ ELISpot. Statistical comparisons employed two-way ANOVA with Bonferroni correction. Symbols indicate individual animals.
